# Trends in Childhood Thyroid Cancer incidence in Korea and Its Potential Risk Factors

**DOI:** 10.3389/fendo.2021.681148

**Published:** 2021-05-14

**Authors:** Jun Park, Hyunju Park, Tae Hyuk Kim, Sun Wook Kim, Hye Won Jang, Jae Hoon Chung

**Affiliations:** ^1^ Division of Endocrinology and Metabolism, Department of Medicine, Thyroid Center, Samsung Medical Center, Sungkyunkwan University School of Medicine, Seoul, South Korea; ^2^ Division of Endocrinology, Department of Medicine, Sahmyook Medical Center, Seoul, South Korea; ^3^ Department of Medical Education, Samsung Medical Center, Sungkyunkwan University School of Medicine, Seoul, South Korea

**Keywords:** childhood, thyroid, cancer, incidence, increase, Korea

## Abstract

**Background:**

Although the incidence of thyroid cancer had been increasing until a few years ago, a decrease has been observed in the last years, probably due to the reduction of the screening tests in Korea. Childhood thyroid cancer has been increasing in the past with the same trend as in adults, but there have been few reports on recent trends. We analyzed the trends of thyroid cancer in Korean children and related factors.

**Methods:**

From national statistics and cancer register database, the data of age-specific incidence rate in Korean childhood thyroid cancer from 1999 to 2017 was obtained, and levels of seaweed intake, the number of computed tomography (CT) and neck ultrasonography (US), obesity prevalence rate, and smoking and alcohol consumption rates in children were analyzed.

**Results:**

The age-specific incidence of thyroid cancer in Korean children has increased in both genders between 1999 and 2017 (2.0 in 1999 vs. 7.2 in 2017, per population of 100,000), especially in the age group of 14-18 years (1.5 in 1999 vs. 5.5 in 2017, per population of 100,000). During the same period, levels of seaweed intake, number of CT scans and neck US, and prevalence of obesity in children increased significantly, while childhood smoking and alcohol consumption rates decreased.

**Conclusion:**

Unlike the adult thyroid cancer in Korea, childhood thyroid cancer continues to increase, and the cause might be accompanied by actual increases due to the environmental factors such as excessive iodine intake, exposure to medical radiation, and increased obesity prevalence as well as the screening effect.

## Introduction

The worldwide incidence of thyroid cancer has been increasing (1, 2). This is mainly due to earlier detection of small sized thyroid carcinomas using high-resolution ultrasonography (US) (3). However, the incidence of larger thyroid cancers is also increasing (1, 4). Therefore, many experts have emphasized that early detection or over-diagnosis cannot completely explain the observed increase in thyroid cancer (1, 4, 5).

The understanding of predisposing genetic factors is increasing (6, 7). Czene et al. has suggested that genetic factors play a major role in the pathogenesis of thyroid cancer and that the population living in East Asia, including Korea, is genetically susceptible to thyroid cancer (7). Environmental factors, such as iodine intake, increased exposure to radiation, and rising rates of obesity may be the potential candidates to explain this phenomenon (8–12).

In the past, the incidence of childhood thyroid cancer had been increasing, although it remained very low. Siegel et al. also reported that annual percentage changes in the incidence of childhood thyroid cancer between 2001 and 2009 were increasing with a range from 4.3% to 6.6% in the United States (13). Our research team previously reported an increasing trend of childhood thyroid cancer in Korea until 2012 and since most children are not screened for thyroid cancer, 72% of childhood thyroid cancers were detected by palpation rather than screening (14). However, some experts stated that the increased incidence of childhood thyroid cancer is due to overdiagnosis by US-based screening (15).

The incidence of thyroid cancer has been decreasing from around 2012 based on the Korea Central Cancer Registry (KCCR), as US screening tests have decreased in Korea (16). The question arises whether the incidence of childhood thyroid cancer has also decreased since 2012 as in adults. Therefore, in the present study, we evaluated the incidence of childhood thyroid cancer in Korea between 1999 and 2017 to investigate its changes after 2012. We also analyzed the trends in iodine intake, medical radiation, and obesity in children and adolescents, which were known to be associated with the development of thyroid cancer by using data from national statistics and cancer register database.

## Materials and Methods

Data from Statistics Korea (KOSTAT) and the KCCR were used to obtain incidences of thyroid cancer, brain tumors, and leukemia in total and childhood populations. The KCCR has been described in a previous study (14). Mid-year total and childhood populations were calculated as arithmetic averages of the resident registration populations on the first and last days of each year in supplementary data.

The total incidence of thyroid cancer by year was calculated as an age-adjusted incidence rate (population by age of resident registration in 2005). The annual incidence of childhood thyroid cancer was calculated as an age-specific incidence rate by dividing the number of newly diagnosed childhood thyroid cancer patients by the number of persons in the same age group (0-18 years). Incidences for brain tumors and leukemia were also calculated as age-specific incidence rates in supplementary data.

The Korea National Health and Nutrition Examination Survey (NHANES) has been conducted every three years since 1998. Since 2007, it has been operated as a regular annual survey conducted by the Korean Ministry of Health and Welfare (MOHW). This survey is administered to people above the age of 1 year in 11,520 households nationwide, and the consumption of each food group is assessed using a questionnaire in 192 districts and 4,416 households nationwide each year. Data on dietary iodine intake was obtained from the NHANES’s standardized daily seaweed intake trend for each food group. Dried kelp is a food ingredient commonly used to make broth in Korea, and if the ingredients were discarded, the food was treated as not comsumed before 2013. Since 2013, as broth foods were added to the survey, the actual nutrient intake was more accurately reflected, The annual trend data for seaweed intake is the result of standardization with the 2005 estimated population to compensate for the impact of age structure differences.

The number of CT scans in children (0-18 years) was identified through CT cases registered in the HIRA database. The age-specific CT scan rate was calculated as the number of childhood CT scans per year divided by the mid-year childhood population from 2007 to 2018. Moreover, since neck US was claimed for insurance from mid-2013, the number of US cases in children (4-18 years) from 2014 to 2017 was identified from the HIRA database.

Data on rates of childhood (13-18 years) obesity prevalence, smoking, and alcohol consumption were based on a Youth Health Behavior Survey, which is a survey of 800 middle and high schools and approximately 75,000 students, conducted by the MOHW form 2005. Childhood obesity prevalence rate is indicated as the percentage of survey respondents who are above the 95th percentile according to the body mass index (BMI) growth chart by age for children and adolescents in 2017. The childhood smoking rate was calculated as the percentage of survey respondents who have smoked more than one day in the last 30 days, and the childhood alcohol consumption rate was calculated as the percentage of survey respondents who have drunk more than one drink in the last 30 days.

## Results

### Trends in Incidence of Childhood Thyroid Cancer in Korea Between 1999 and 2017

**Figure 1:**
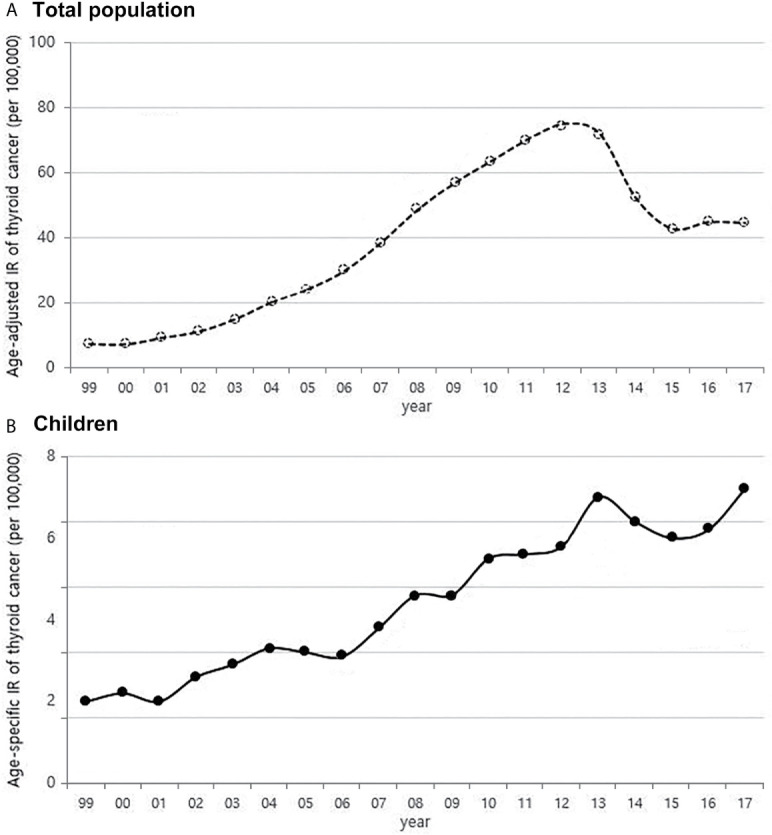
Age-adjusted incidence of thyroid cancer in Korea **(A)** and age-specific incidence rate of childhood thyroid cancer (0-18 years) **(B)** (1999-2017, per population of 100,000). IR, incidence rate.

**Figure 2:**
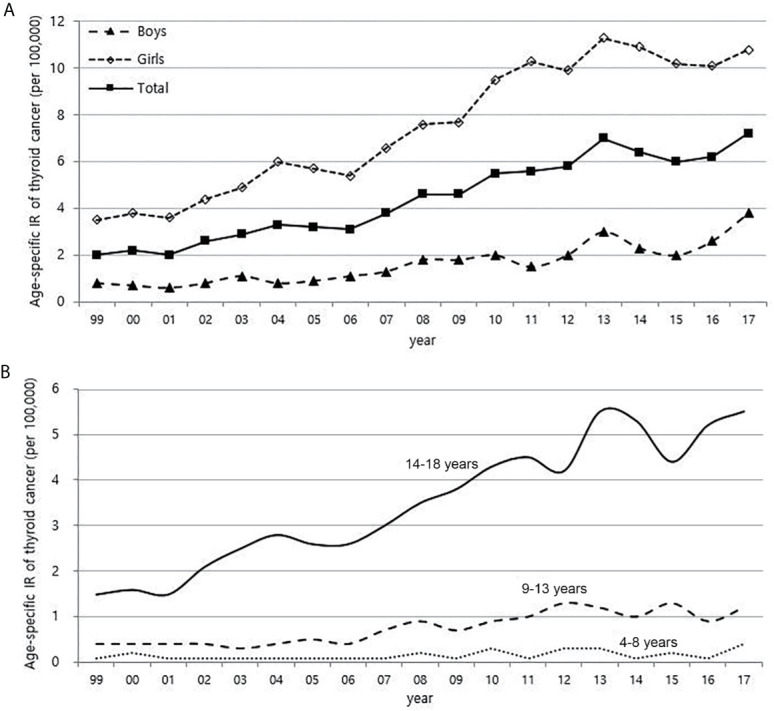
Age-specific incidence rate of childhood (0-18 years) thyroid cancer according to gender **(A)** and age **(B)** (1999-2017, per population of 100,000). IR, incidence rate.

From 1999 to 2017, the number of children with thyroid cancer per year ranges from 79 to 225. The age-adjusted incidence of thyroid cancer in Korea had gradually increased and peaked in 2012, but has been rapidly declining since 2013. The age-specific incidence of childhood (0-18 years) thyroid cancer in Korea also increased and peaked in 2013, followed by a decrease. However, it has been increasing again since 2015 (**Figure 1**). The age-specific incidence of childhood thyroid cancer saw a 3.6-fold increase from 2.0 per 100,000 children in 1999 to 7.2 per 100,000 children in 2017. As in adult, girls have higher incidence rates of thyroid cancer than boys, but while the rate of increase in incidence among girls was faster until 2012, the rate of increase in incidence in boys became faster from 2012 to 2017. Between 1999 and 2017, the 4.75-fold overall increase in the incidence rate in boys (0.8 vs. 3.8 per 100,000, respectively) was larger than the 3.1-fold increase seen in girls (3.5 vs. 10.8 per 100,000, respectively) (**Figure 2A**). According to the age-specific incidence rates, the age group of 14-18 years accounted for the majority, approximately 75%, of all childhood thyroid cancer cases, and this group’s rate continued to increase from 1.5 per 100,000 in 1999 to 5.5 per 100,000 in 2017 (**Figure 2B**).

### Environmental Factors That Might Result in an Increase in Childhood Thyroid Cancer

**Figure 3:**
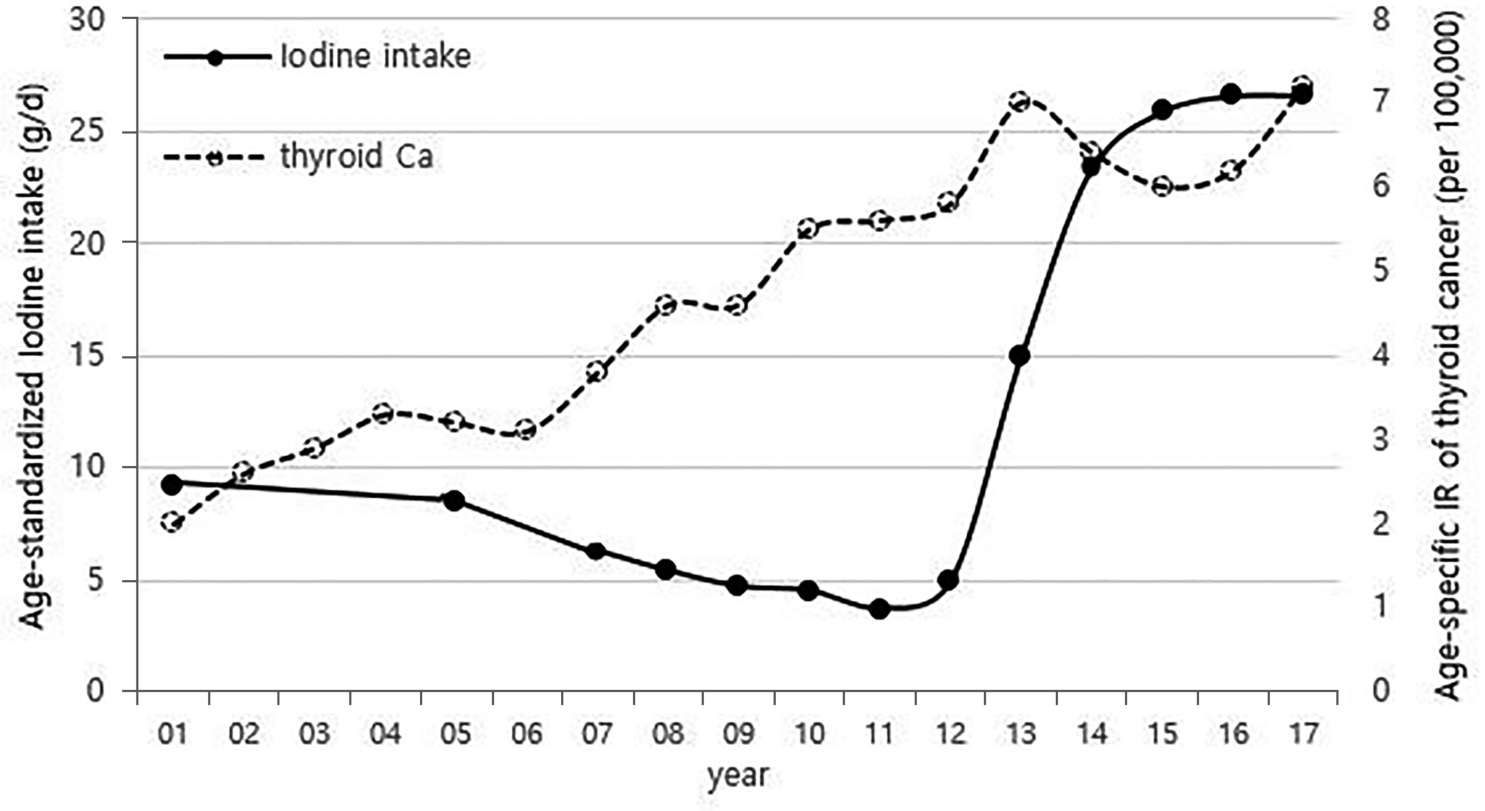
Correlation between age-standardized iodine intake (2007-2017) and age-specific incidence rate of childhood thyroid cancer (0-18 years) by year in Korea (1999-2017, per population of 100,000). Broth foods were added to the survey since 2013. Ca, cancer; IR, incidence rate.

**Figure 4:**
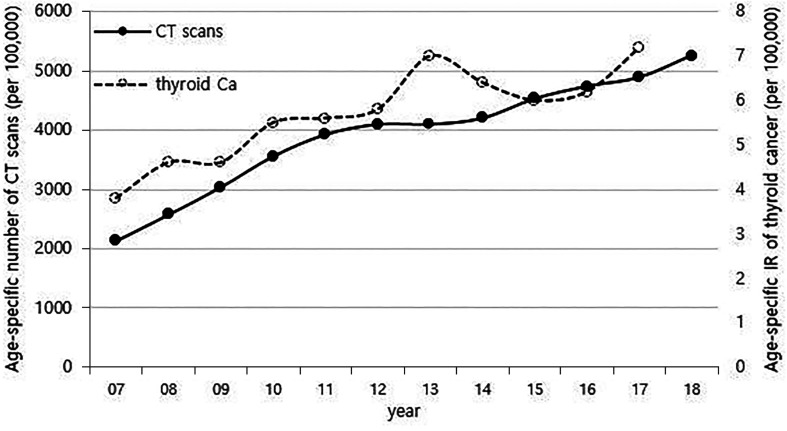
Correlation between the age-specific number of CT scans in childhood (0-18 years) (2007-2018, per population of 100,000) and age-specific incidence rate of childhood thyroid cancer (0-18 years) by year in Korea (2007-2017, per population of 100,000). CT, computed tomography; Ca, cancer; IR, incidence rate.

**Figure 5:**
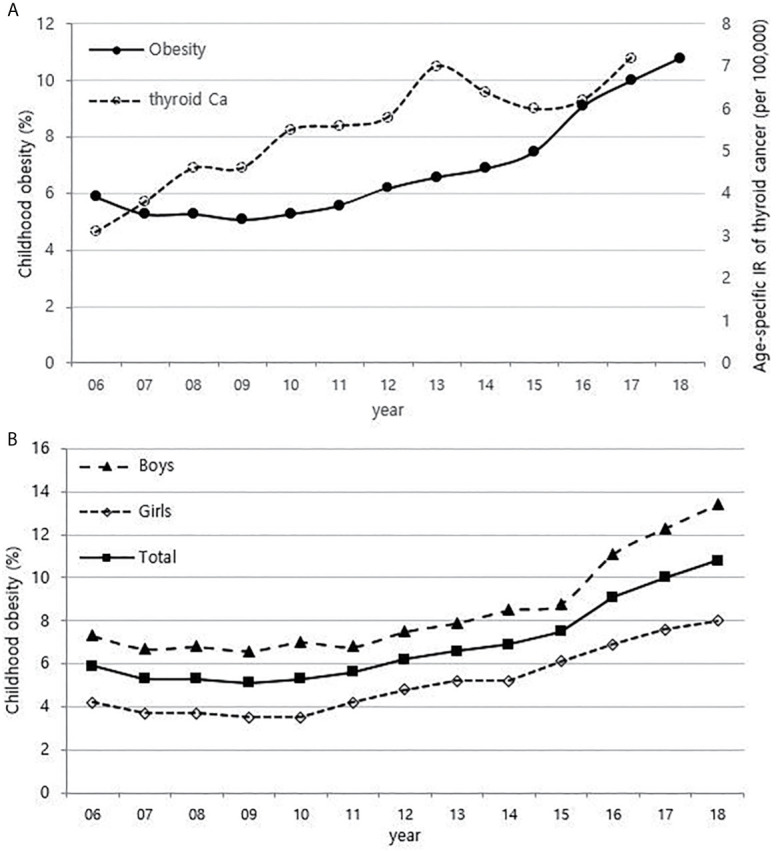
Correlation between childhood obesity prevalence (13-18 years) (2006-2018) and age-specific incidence rate of childhood thyroid cancer (0-18 years) by year in Korea (2006-2017, per population of 100,000) **(A)**. Childhood obesity prevalence according to sex **(B)**. Ca, cancer; IR, incidence rate.

**Figure 6:**
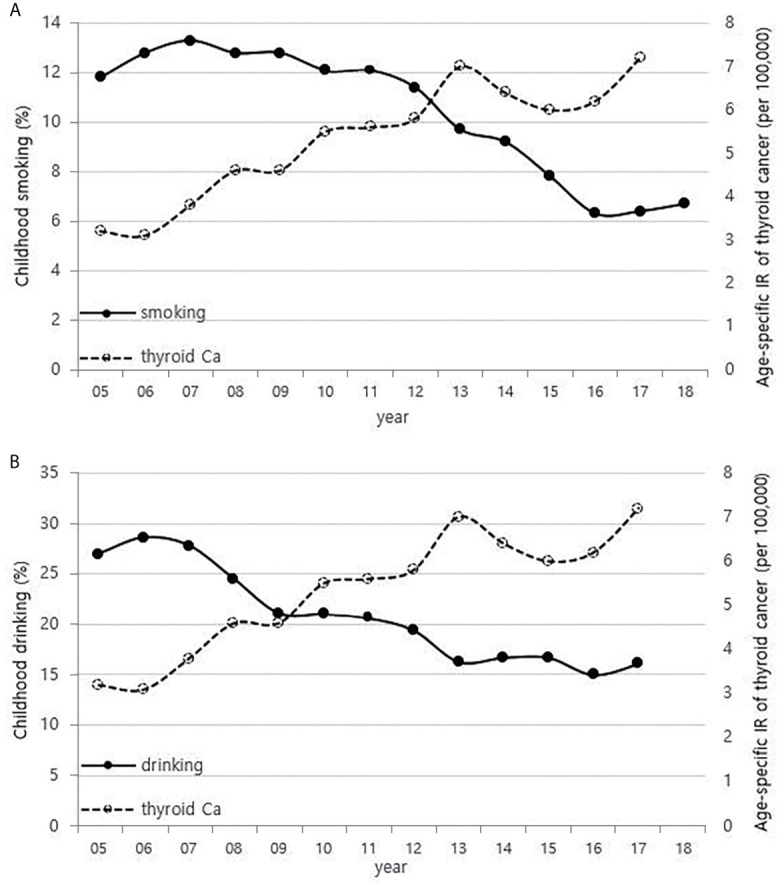
Correlation between childhood smoking **(A)** and drinking **(B)** rates (13-18 years) (2005-2018) and age-specific incidence rate of childhood thyroid cancer (0-18 years) in Korea (2005-2017, per population of 100,000). Ca, cancer; IR, incidence rate.

Although it is not possible to compare before 2013 (broth food was not included), but the age-standardized rates of seaweed intake, which accounts for the bulk of dietary iodine consumption in Korea, have risen since 2013 when broth foods were added to the survey, based on the Korea NHANES (**Figure 3**). The number of CT scans in children and adolescents (0-18 years) has been increasing every year from approximately 260,000 cases in 2007 to 490,000 cases in 2018 (**Figure 4**). Adolescent (13-18 years) obesity rates from 2006 to 2018 also showed a steady increase in both genders (from 5.9% in 2006 to 10.8% in 2018), which corresponds to the increase in incidence of childhood thyroid cancer (**Figures 5A, B**). On the other hand, adolescent (13-18 years) cigarette smoking and alcohol consumption rates tended to decrease from 2005 to 2018 (11.8% to 6.7% and 27.0% to 16.1%, respectively), corresponding to a negative correlation with the incidence of childhood thyroid cancer (**Figures 6A, B**).

### Trends in Neck US Exams in Korean Children Between 2014 and 2018

**Figure 7:**
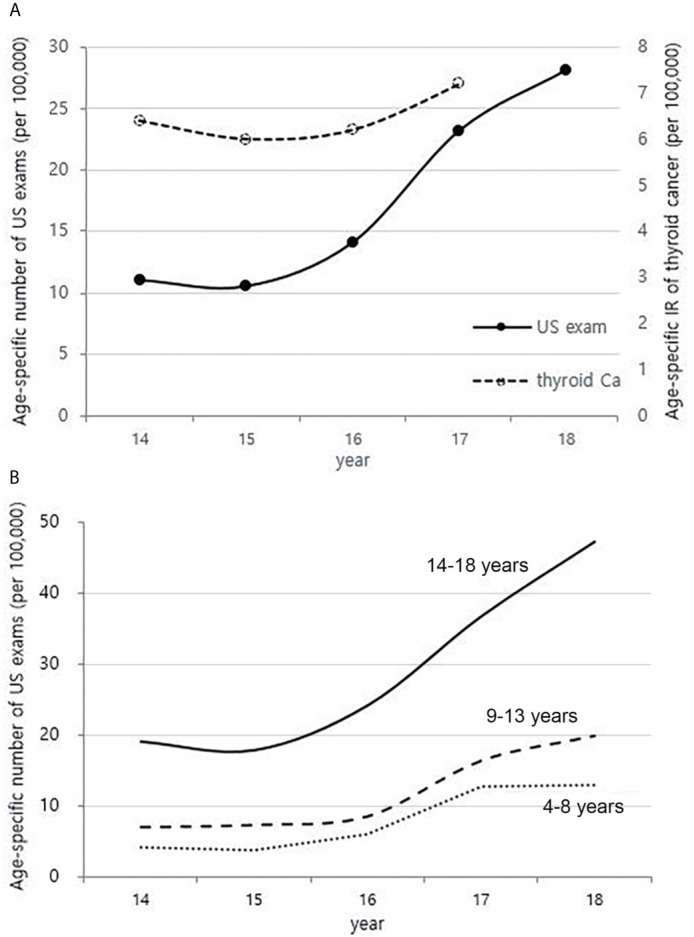
Correlation between the age-specific number of neck US exams in childhood (4-18 years) (2014-2018, per population of 100,000) and age-specific incidence rate of childhood thyroid cancer (0-18 years) by year in Korea (2014-2017, per population of 100,000) **(A)**. Age-specific number of neck US exams in childhood (4-18 years) according to age groups (2014-2018, per population of 100,000) **(B)**. US, ultrasonography; Ca, cancer; IR, incidence rate.

The national health insurance guarantee of neck US has begun from mid-2013, and the age-specific number of US exams for children charged with HIRA from 2014 to 2018 has increased sharply in all age groups (**Figures 7A, B**). This is different from the overall trend in incidence of childhood thyroid cancer, especially those aged 14-18 years (**Figures 7B** and **2B**).

## Discussion

We investigated trends in the incidence of childhood thyroid cancer in Korea over the past 18 years and also analyzed the trends in previously considered potential risk factors for thyroid cancer, such as the dietary iodine intake, the number of CT scans performed, and obesity prevalence in children or adolescents, by using data representing the Korean population.

Several studies have shown that iodine intake levels that are lower or higher than recommended result in an increase in thyroid cancer. Mousavi et al. showed that there were high rates of thyroid cancer among first-generation immigrants to Sweden from areas with inadequate or excessive levels of iodine intake (17). Michikawa et al. demonstrated that the incidence of thyroid cancer was significantly higher in Japanese women, who consumed seaweed more than 3-4 times per week, than in those who consumed seaweed fewer than 2 days per week, with hazard ratio 1.71 (95% CI: 1.01-2.90, *p* = 0.04) (18). In fact, there is a high level of iodine consumption in Korean due to the large amounts of seaweed intake (19). Iodine consumption in Korean children is higher than in adult groups (**Figure S1**) (20), and children have a relatively smaller volume of thyroid gland than adults, thus, it is expected that high iodine intake can have a greater impact on the development of thyroid cancer in children.

Radiation exposure of the thyroid, particularly in childhood, is a clearly established risk factor for the development of thyroid cancer, as demonstrated by the Chernobyl accident (21). There is a report that there was no significant increase in the incidence of pediatric thyroid cancer after the Fukushima Daiichi Nuclear Power Plant accident (22), but considering that the incidence of thyroid cancer increased rapidly after 4-5 years at the time of the Chernobyl accident, it is necessary to understand the latency phase. CT scans have been the largest contributor to radiation exposure in recent years. The childhood population in Korea steadily decreased (**Figure S2**), while the age-specific number of CT scans has steadily increased since 2007. The use of CT scans is rising more rapidly in the childhood population than in adults, and the thyroid glands of children are more sensitive to radiation than those of adults (23, 24). It is known that CT scans in children also increase the risk of developing brain tumors and leukemia as well as thyroid cancer, and the increase in risk is greatest for brain tumors (25, 26). However, while the incidence of childhood thyroid cancer is rapidly increasing, the incidence of brain tumors and leukemia in children has only been minimally increasing during the same period (**Figure S3**). Thus, the recent increase in childhood thyroid cancer in Korea may be associated with the increase in medical exposure, but it is not expected to be a major factor. In addition, as neck US exam of children has been also increasing recently (**Figure 7**), there must be some portion of the increase due to the incidental detection of small sized thyroid cancer. However, the increase in childhood thyroid cancer cannot be fully explained by the increase in incidental detection, and the true increase should be taken into account.

The relationship between obesity and thyroid cancer is still controversial, but there seems to be a positive association between BMI and the incidence of thyroid cancer, particularly in women (9, 27, 28). Thyroid cancer is almost three times more common in women than in men, and it has been suggested that the female sex hormones, especially estrogen, are related (29). Estrogen receptors are found in thyroid tissues. Therefore, the thyroid glands of women are more likely to be irritated by estrogen than those of men, causing inflammation and various thyroid diseases. There are also reports that estrogen promotes the proliferation of thyroid cancer cells (30, 31). In recent years, precocious puberty has been increasing with childhood obesity, and obesity also increases insulin resistance and inhibits sex hormone-binding proteins, which results in an increase in estrogen levels (32, 33). Since the prevalence of childhood obesity in Korea is rapidly increasing, exposure to estrogen at an early age might be contributing to the increase in childhood thyroid cancer. In particular, the prevalence of obesity in boys has been increasing more rapidly than in girls since 2015 in **Figure 2A** of this study, and this may be related to the recent steeper increase in the incidence of thyroid cancer in boys than in girls.

Cigarette smoking and alcohol consumption has an inverse association with thyroid cancer incidence. Many researchers have proposed that since higher thyroid-stimulating hormone (TSH) levels are associated with higher frequency and advanced stage of thyroid cancer (34), and low TSH levels are measured in smokers, suggesting that smokers have a lower risk of thyroid cancer (35, 36). Cho et al. found that men who are currently smoking had a lower risk of incident thyroid cancer even after adjusting for TSH and BMI in a cohort study of 96,855 Korean adults (37), and the reason is probably due to the anti-estrogenic effect of smoking (9, 35, 38). A meta-analysis showed that alcohol intake is responsible for reducing the risk of thyroid cancer (39), and Meinhold et al. demonstrated in a large prospective study that people who consume more than two alcoholic drinks a day have a reduced risk of thyroid cancer compared to those who do not drink alcohol (relative risk = 0.57, 95% CI 0.36–0.89, *p*-trend = 0.01) (40).

When considering the results from previous research and the positive correlations in overall trend between the incidence of thyroid cancer and dietary iodine consumption, the number of CT scans, and obesity prevalence that were observed among children in this study (**Figures 1**, **3**–**5**), we might be tempted to suggest that when there is a genetic susceptibility, excessive iodine, medical radiation, and obesity are likely to cause thyroid cancer by acting as driving factors in children.

There are several limitations in this study. First, since this research is an ecological study of the entire population, it is not known whether the individual children who were diagnosed with thyroid cancer actually had a high dietary iodine intake, underwent several CT scans, or gained weight. Therefore, the results cannot be interpreted at an individual level, but hypotheses for the next step of research have been presented. Second, considering the potentially long latency periods between exposure and disease onset, it was recommended to refer to the amount of dietary iodine intake, the number of childhood CT scan use, and the childhood obesity prevalence data before 2005. However, since such earlier data were not available, it is meaningful to keep track of the trend of childhood thyroid cancer after 2017. Third, this analysis is the lack of data on tumor detection methods and characteristics such as histology, stage, and size at diagnosis. In addition, since the increase in childhood thyroid cancer is mainly seen in the population of 14-18 years, further analysis of this age group would be more informative, but there was a limit to further analysis as only the nationwide data already collected and published were used. Nevertheless, because there is not enough research on the development of childhood thyroid cancer, the comparison of childhood thyroid cancer incidence trends with total thyroid cancer incidence and the analysis of trends in prevalence of potential risk factors during the same period are a potentially valuable contribution.

In conclusion, the incidence of childhood thyroid cancer is constantly increasing. Although the number of thyroid US exams in children is also increasing, it cannot be concluded that the increase is only due to the screening effect. In addition, we cannot rule out the possibility that a true increase due to the potential risk factors may be implied. Since the environmental factors that can reduce or prevent childhood thyroid cancer can be overlooked, further studies are needed on the causes of the increase in childhood thyroid cancer.

## Data Availability Statement

The original contributions presented in the study are included in the article/**Supplementary Material**. Further inquiries can be directed to the corresponding author.

## Ethics Statement

The studies involving human participants were reviewed and approved by Institutional Review Board in Samsung Medical Center (SMC-IRB number: 2020-04-041). Written informed consent from the participants’ legal guardian/next of kin was not required to participate in this study in accordance with the national legislation and the institutional requirements.

## Author Contributions

JP conceptualized and designed the study, drafted the initial manuscript, and reviewed and revised the manuscript. HP designed the data collection instruments, collected data. TK, SK, and HJ reviewed and revised the manuscript. JC conceptualized and designed the study, coordinated and supervised data collection, and critically reviewed the manuscript for important intellectual content. All authors contributed to the article and approved the submitted version.

## Funding

This work was supported by the Samjung Scholarship Foundation.

## Conflict of Interest

The authors declare that the research was conducted in the absence of any commercial or financial relationships that could be construed as a potential conflict of interest.
