# Supplementary material for: Trends in Childhood Thyroid Cancer incidence in Korea and Its Potential Risk Factors
Source: Front Endocrinol (Lausanne). 2021 May 14;12:681148. doi: 10.3389/fendo.2021.681148 (PMC8160442; doi:10.3389/fendo.2021.681148)
Supplement: Supplementary file 1 [file DataSheet_1.doc]

***Supplementary Material***


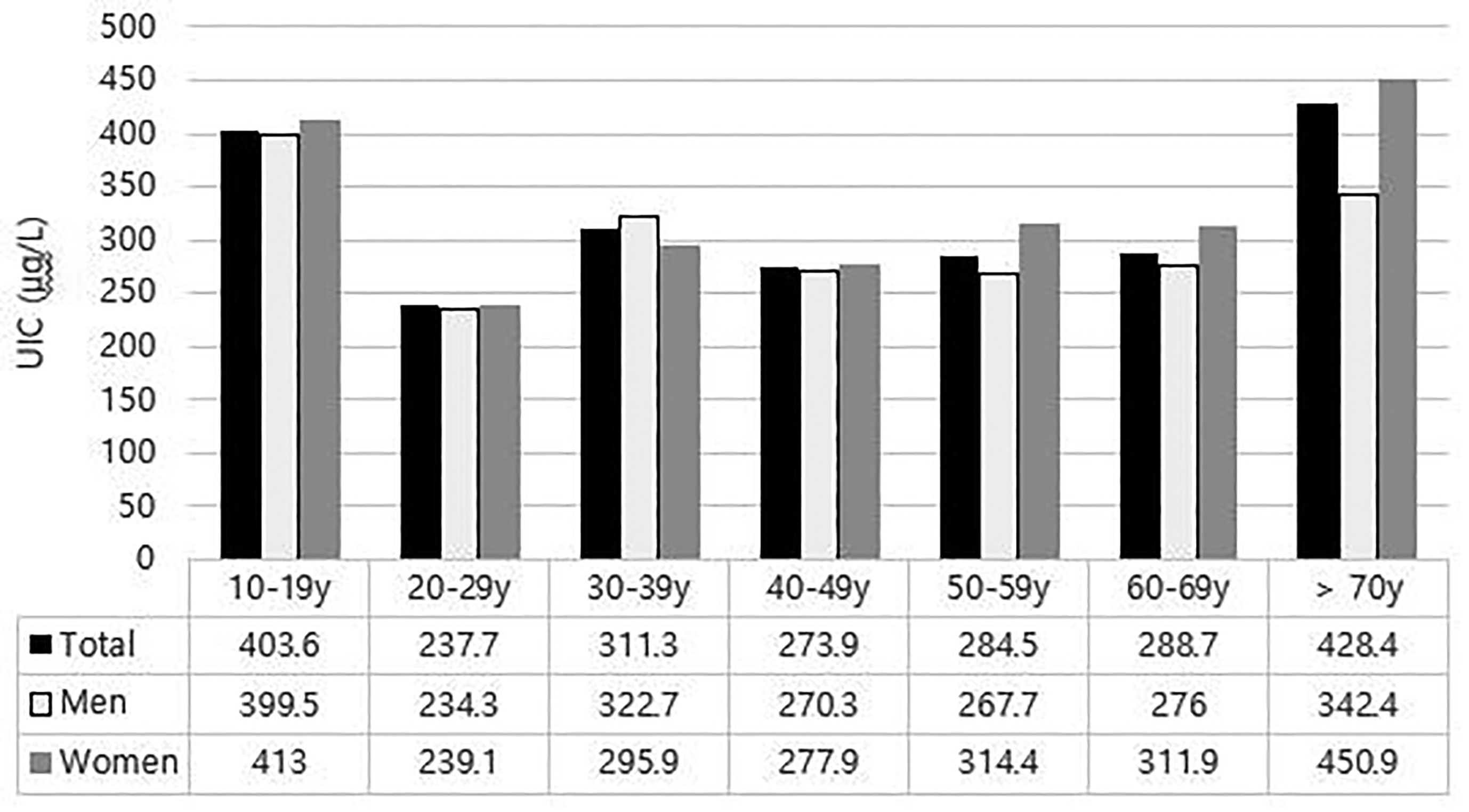


Supplementary Figure 1. Urinary iodine concentration (UIC, μg/L) by age and sex. The median UIC was higher in school-aged children and in the above 70 years age group than in other age groups. Adapted from Park *et al.* (20). *Abbreviations*: UIC, urinary iodine concentration; y, years.


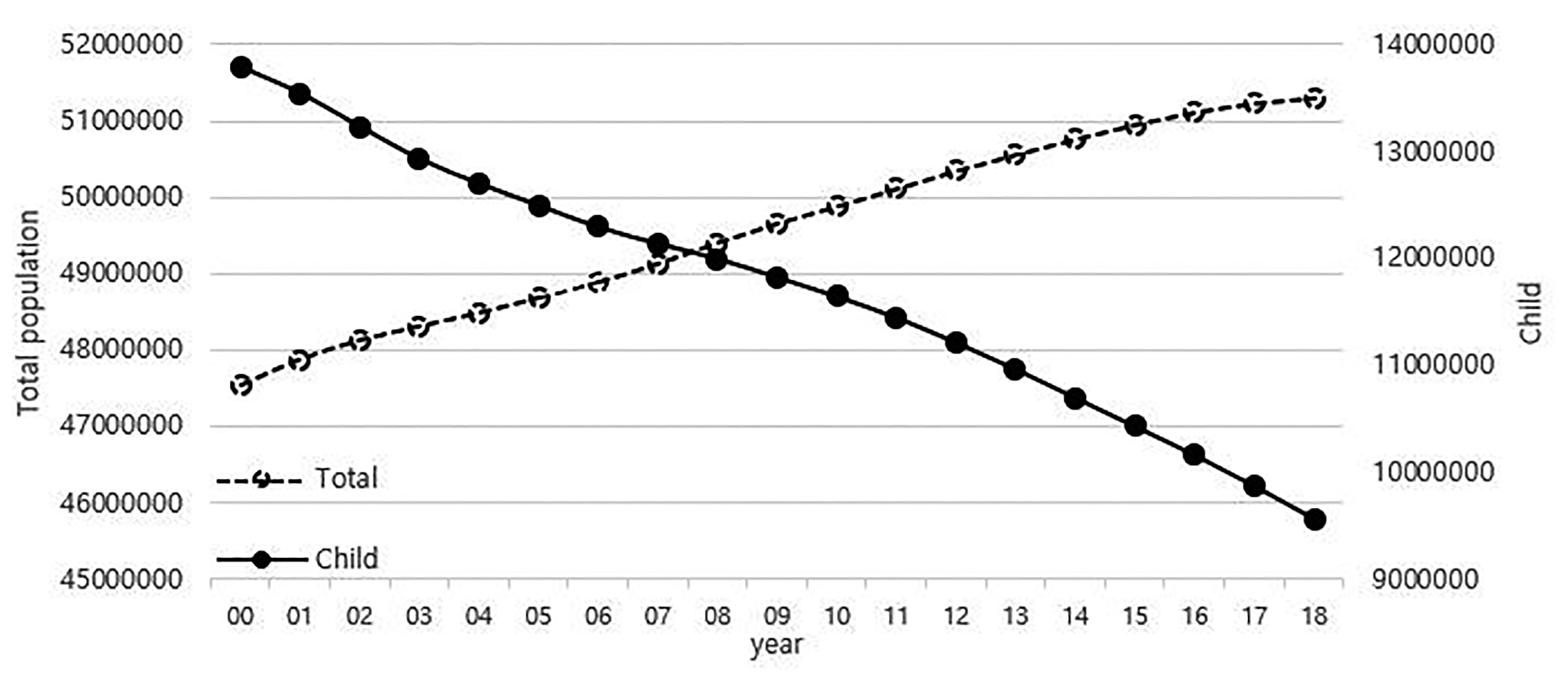


Supplementary Figure 2. Change in total and childhood population in Korea from 2000 to 2018.


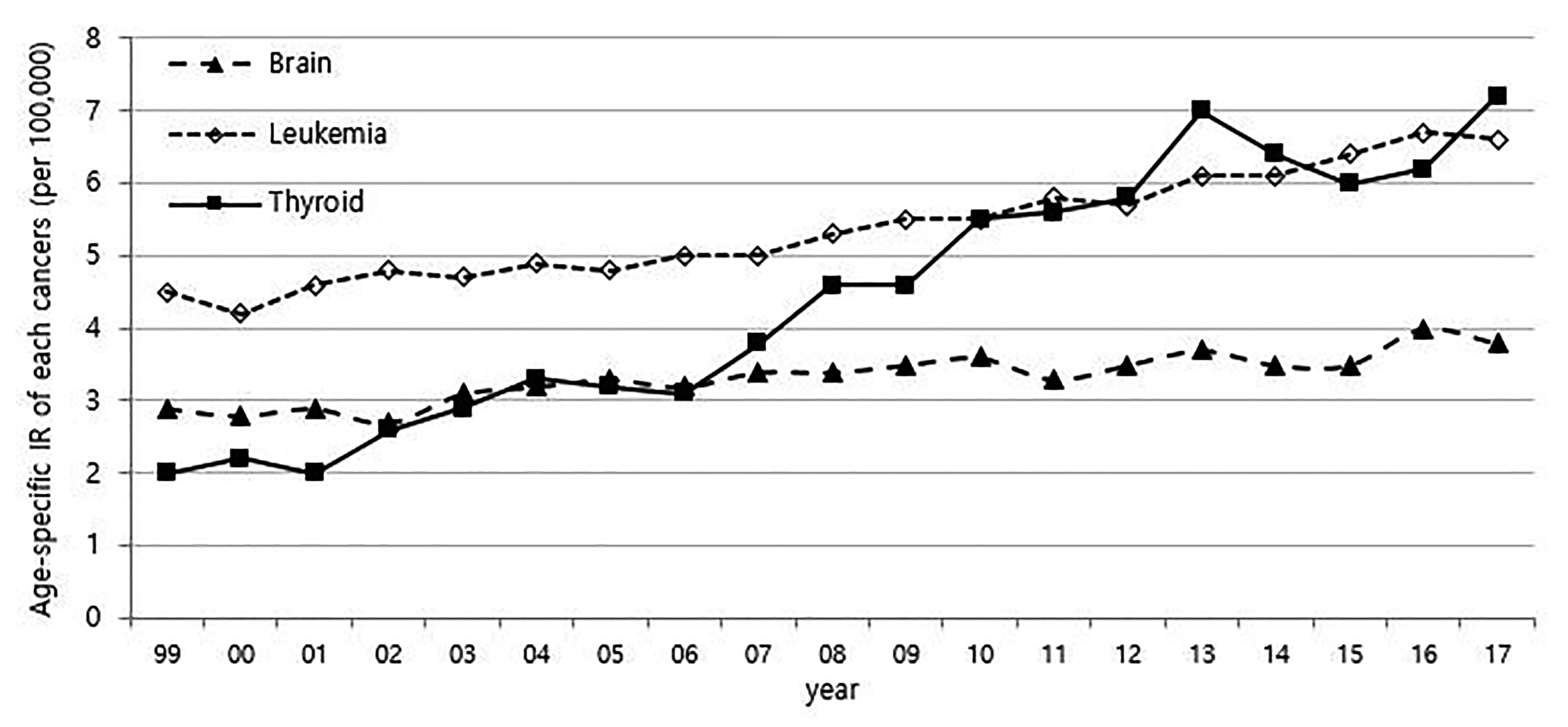


Supplementary Figure 3. Age-specific incidence rate of thyroid cancer, brain tumor, and leukemia in childhood (0-19 years). (1999-2017, per population of 100,000). *Abbreviations*: IR, incidence rate.
